# Epigallocatechin-3-Gallate Promotes Osteo-/Odontogenic Differentiation of Stem Cells from the Apical Papilla through Activating the BMP–Smad Signaling Pathway

**DOI:** 10.3390/molecules26061580

**Published:** 2021-03-12

**Authors:** Zeni Liu, Yuxiu Lin, Xiaolin Fang, Jingwen Yang, Zhi Chen

**Affiliations:** The State Key Laboratory Breeding Base of Basic Science of Stomatology (Hubei-MOST) & Key Laboratory of Oral Biomedicine Ministry of Education, School and Hospital of Stomatology, Wuhan University, Wuhan 430079, China; zeni@whu.edu.cn (Z.L.); 2016103040012@whu.edu.cn (Y.L.); xlfang@whu.edu.cn (X.F.)

**Keywords:** stem cells from apical papilla, epigallocatechin-3-gallate, osteo-/odontogenic differentiation, proliferation, BMP–Smad signaling pathway

## Abstract

Stem cells from apical papilla (SCAPs) are desirable sources of dentin regeneration. Epigallocatechin-3-gallate (EGCG), a natural component of green tea, shows potential in promoting the osteogenic differentiation of bone mesenchymal stem cells. However, whether EGCG regulates the odontogenic differentiation of SCAPs and how this occurs remain unknown. SCAPs from immature human third molars (16–20 years, *n* = 5) were treated with a medium containing different concentrations of EGCG or bone morphogenic protein 2 (BMP2), with or without LDN193189 (an inhibitor of the canonical BMP pathway). Cell proliferation and migration were analyzed using a CCK-8 assay and wound-healing assay, respectively. Osteo-/odontogenic differentiation was evaluated via alkaline phosphatase staining, alizarin red S staining, and the expression of osteo-/odontogenic markers using qPCR and Western blotting. We found that EGCG (1 or 10 μM) promoted the proliferation of SCAPs, increased alkaline phosphatase activity and mineral deposition, and upregulated the expression of osteo-/odontogenic markers including dentin sialophosphoprotein (*Dspp*), dentin matrix protein-1 (*Dmp-1*), bone sialoprotein (*Bsp*), and Type I collagen (*Col1*), along with the elevated expression of BMP2 and phosphorylation level of Smad1/5/9 (*p* < 0.01). EGCG at concentrations below 10 μM had no significant influence on cell migration. Moreover, EGCG-induced osteo-/odontogenic differentiation was significantly attenuated via LDN193189 treatment (*p* < 0.01). Furthermore, EGCG showed the ability to promote mineralization comparable with that of recombinant BMP2. Our study demonstrated that EGCG promotes the osteo-/odontogenic differentiation of SCAPs through the BMP–Smad signaling pathway.

## 1. Introduction

Irreversible pulpitis and pulp necrosis in developing teeth usually lead to the formation of nonvital pulp tissue and the ceasing of root development. The emergence of regenerative endodontics provides a promising approach to treating these diseases [1,2,3]. Stem cells from apical papilla (SCAPs) are a population of cells existing in the root apex of the immature tooth, contributing to root dentin formation and root elongation during root maturation [4,5]. SCAPs exhibit great viability and potency of multipotent differentiation, including odontogenic, osteogenic, neurogenic, and adipogenic differentiation [6,7,8]. Furthermore, SCAPs, along with periodontal ligament stem cells (PDLSCs), can regenerate a bio-root with periodontal ligament in vivo [6]. Therefore, SCAPs are considered to be desirable cell sources in regenerative dentistry.

Green tea is one of the most commonly consumed drinks around the world. Epigallocatechin-3-gallate (EGCG) is the major functional component and the most abundant catechin polyphenol in green tea. EGCG has attracted great attention because it is easy to obtain and has potent biological effects, such as anti-inflammatory, antibacterial, antioxidative, and anticarcinogenic activities [9]. Previous studies have reported that EGCG improves the osteogenic differentiation of human bone mesenchymal stem cells (BMSCs) [10], human periodontal ligament cells [11], and human adipose-derived stem cells [12] in vitro. EGCG modified scaffolds enhance osteogenesis and improve bone healing in vivo [13,14]. A recent study also revealed that EGCG enhances the odontogenic differentiation of dental pulp stem cells (DPSCs) [15]. According to the abovementioned facts, EGCG seems to be a good agent for promoting osteo-/odontogenic differentiation and mineralized tissue formation. Additionally, EGCG exhibits a great ability to promote the proliferation of human bone mesenchymal stem cells [16] as well as neural stem cells [17,18]. However, the effects of EGCG on the proliferation and osteo-/odontogenic differentiation of SCAPs are unknown.

Odontoblast differentiation and tooth root formation are regulated by several signaling pathways, such as the Wnt signaling pathway and the BMP–Smad signaling pathway [4]. Several BMPs, such as BMP2 and BMP7, are expressed during odontoblast differentiation [19]. Moreover, the controlled release of BMP2 promotes dentin formation from SCAPs in vivo [20]. However, it is unclear whether the BMP–Smad signaling pathway is required for the osteo-/odontogenic differentiation of SCAPs during EGCG treatment.

In this study, we aimed to determine the effects of EGCG on SCAPs by detecting cell proliferation, migration, and odontogenic differentiation, and investigated how EGCG regulates the osteo-/odontogenic differentiation of SCAPs. The null hypothesis of our study was EGCG influences the proliferation, migration, and odontogenic differentiation of SCAPs, and EGCG affects the odontogenic differentiation of SCAPs through BMP signaling. Our results revealed that EGCG can promote the proliferation and osteo-/odontogenic differentiation of SCAPs by activating the BMP–Smad signaling pathway. Furthermore, we found that EGCG showed odontogenic capacity similar to that of recombinant BMP2 in vitro. These findings indicate that EGCG can be a promising natural compound to facilitate dentin regeneration.

## 2. Results

### 2.1. In Vitro Characterization of SCAPs

**Figure 1:**
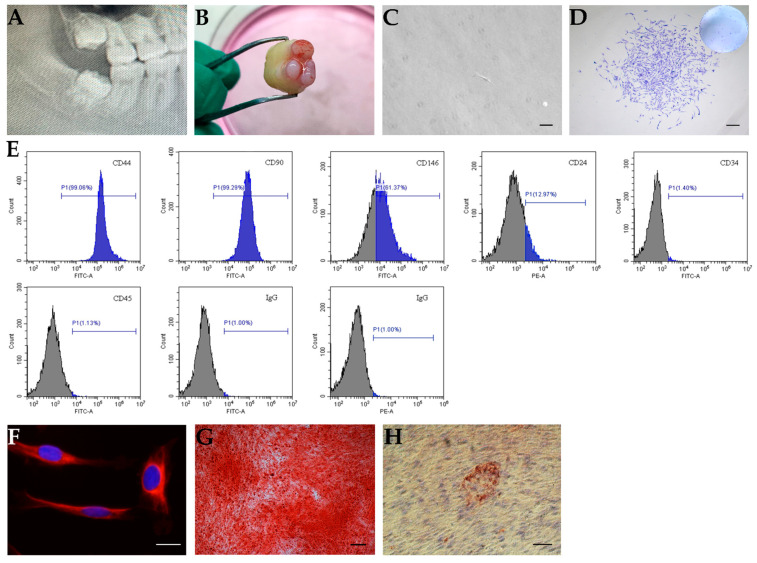
In vitro characterization of stem cells from apical papilla (SCAPs). (**A**,**B**) The apical papilla tissue from an immature human tooth. (**A**) A representative radiograph indicating the third molar with the open apex; (**B**) apical papilla tissue. (**C**,**D**) The colony formation of SCAPs. (**C**) SCAPs exhibited a typical spindle shape, scale bar = 100 μm. (**D**) Isolated cells formed colony-like units, scale bar = 200 μm. (**E**) Flow cytometric analysis of SCAPs using several markers. Cells were positive for CD44, CD90, CD146, and CD24, and negative for CD34 and CD45. (**F**) Immunofluorescence staining showed nestin expression in SCAPs, scale bar = 20 μm. (**G**) SCAPs formed mineralized nodules after osteo-/odontogenic differentiation, scale bar = 200 μm. (**H**) SCAPs were differentiated into adipocytes with Oil Red O-positive lipid clusters when cultured in adipogenic differentiation medium for four weeks, scale bar = 50 μm.

To obtain SCAPs, we obtained apical papilla from immature human third molars indicated by radiograph (Figure 1A,B) and isolated SCAPs. The isolated cells exhibited colony-like growth after 10 days of culture (Figure 1C,D). Cells expressed several cell surface markers, such as CD44 (99.06%), CD90 (99.29%), CD146 (61.37%), and CD24 (12.97%) (Figure 1E), whereas cells were negative for the hematopoietic linage markers CD34 and CD45 (Figure 1E). Additionally, the cells positively expressed the neurogenic marker Nestin, which indicated the SCAPs may be from a neural crest origin of mesenchymal stem cells (MSCs) (Figure 1F). In addition, after induction in adipogenic or osteo-/odontogenic culture medium, the isolated cells formed extensive mineral deposits or lipid clusters, respectively (Figure 1G,H).

### 2.2. The Effects of EGCG on Proliferation and Migration of SCAPs

**Figure 2:**
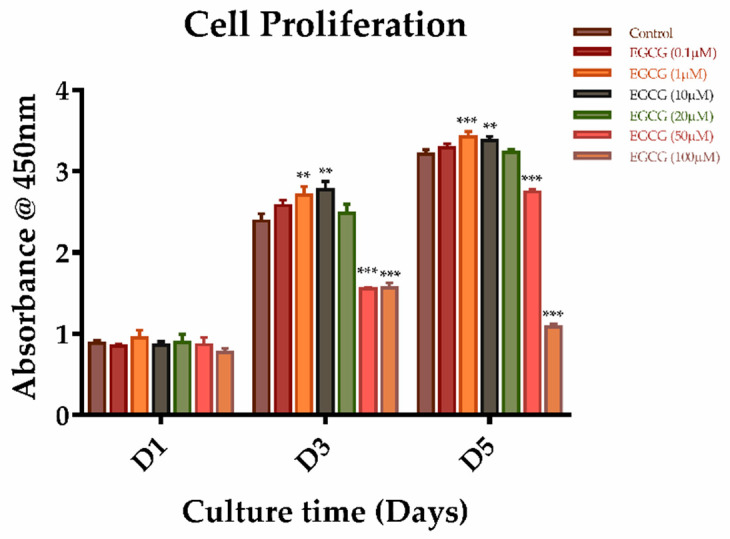
The effects of different concentrations of epigallocatechin-3-gallate (EGCG) on the proliferation of SCAPs. A graph depicting the proliferation of SCAPs treated with different doses of ECGC at days 1, 3, and 5 using a CCK-8 assay (*n* = 5). (** *p* < 0.01, *** *p* < 0.001). Error bars: mean± standard deviation.

**Figure 3:**
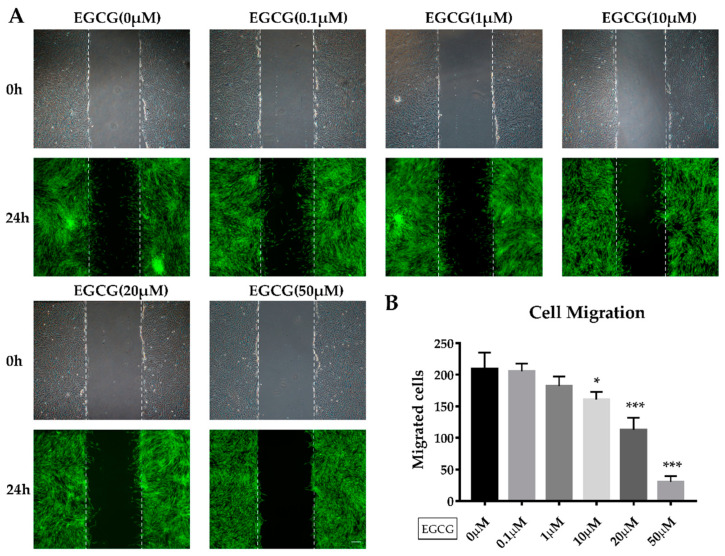
The migration of SCAPs treated with different doses of EGCG. (**A**) The representative images of the wound-healing assay were obtained at 0 and 24 h after treatment with different concentrations of EGCG. (**B**) Migrated cells were counted in each group after 24 h. EGCG at low concentrations exhibited no significant effect compared with the control group, while over 10 μM of EGCG inhibited the migration of SCAPs. Scale bar = 200 μm. (* *p* < 0.05, *** *p* < 0.001). Error bars: mean± standard deviation.

To determine the effects of EGCG on the proliferation and migration of SCAPs, cells were treated with EGCG at different concentrations. We found that EGCG at concentrations of 0.1–100 μM showed no significant effect on the proliferation of SCAPs on day 1. EGCG treatment at 1 or 10 μM increased the proliferation of SCAPs at days 3 and 5 when compared with the control group. However, cells showed a significantly lower proliferation ability when treated with 50 or 100 μM EGCG after three and five days of culture (Figure 2). We also determined the effect of EGCG on the migration of SCAPs using a wound healing assay. Although higher concentrations of EGCG (≥10 μM) significantly attenuated the migration of SCAPs, low concentrations of EGCG (0.1 or 1 μM) did not affect the migration of SCAPs after 24 h of incubation (Figure 3A,B). Based on these results, we used EGCG concentrations no higher than 10 μM for the following experiments.

### 2.3. EGCG Promotes Osteo-/Odontogenic Differentiation and Mineralization of SCAPs

**Figure 4:**
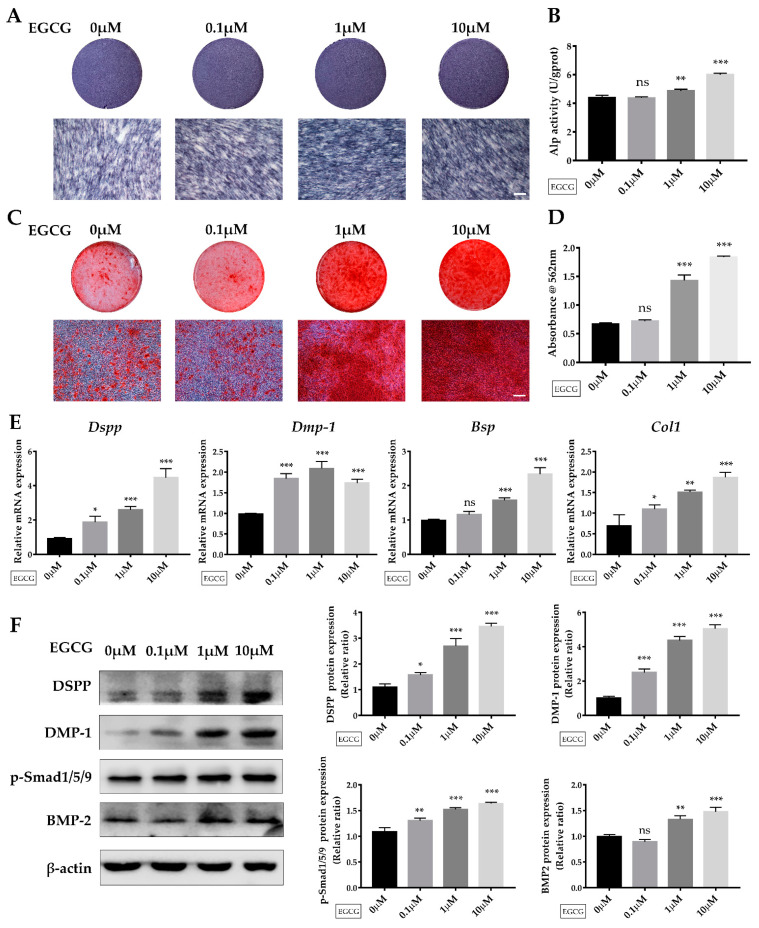
The role of EGCG on the osteo/odontogenic differentiation of SCAPs. (**A**) ALP staining of SCAPs in each group after seven days of osteo-/odontogenic induction. (**B**) ALPase activity results showed the upregulation of ALP in EGCG-treated groups at day 7. (**C**) Alizarin red S staining indicated the formation of mineralized nodules after 14 days of osteo-/odontogenic induction. (**D**) Semi-quantification of calcium deposits after 14 days of induction. (**E**) The expression of *Dspp*, *Dmp-1*, *Bsp*, and *Col1* was detected at 14 days (*n* = 3). (**F**) The protein expression and grayscale analysis of DSPP, DMP-1, and BMP2 and the phosphorylation level of Smad1/5/9 at 14 days. Scale bar = 100 μm. (* *p* < 0.05, ** *p* < 0.01, *** *p* < 0.001, ns: not statistically significant). Error bars: mean ± standard deviation. Dspp: dentin sialophosphoprotein; Dmp-1: dentin matrix protein-1; Bsp: bone sialoprotein; Col1: Type I collagen.

To evaluate the effect of EGCG on the osteo-/odontogenic differentiation and mineralization of SCAPs, cells were cultured in induction medium containing 0, 0.1, 1, or 10 μM EGCG for seven or 14 days. After seven days of induction, ALP staining and ALP activity revealed that 1 or 10 μM EGCG enhanced the ALP level of SCAPs (Figure 4A,B).

Then, extracellular mineral deposits were determined by ARS staining (Figure 4C) and semi-quantitative analysis (Figure 4D) after 14 days of induction. We determined that treatment with 1 or 10 μM EGCG significantly promoted the formation of extracellular mineral deposits, although cells treated with 0.1 μM EGCG exhibited comparable results with the control group. Dentin sialophosphoprotein (*Dspp*), one of the critical odontogenic markers, was significantly enhanced in EGCG-treated groups on day 14 compared with the control group (Figure 4F). With the addition of EGCG, the mRNA level of dentin matrix protein-1 (*Dmp-1*) and type I collagen (*Col 1*) was also elevated. EGCG treatment at 1 or 10 μM significantly enhanced the expression levels of bone sialoprotein (*Bsp*), although the 0.1 μM EGCG-treated group showed an effect on *Bsp* expression that was comparable with the control group after 14 days of induction. Cells treated with 10 μM EGCG exhibited the most remarkable increases in the expression levels of *Dspp*, *Bsp*, and *Col 1* on day 14. These changes were further confirmed by western blot analysis. As shown in Figure 4F, the protein levels of osteo-/odontogenic markers DSPP and DMP-1 were significantly enhanced via EGCG treatment. Collectively, these results indicated that 1 or 10 μM EGCG promotes the osteo-/odontogenic differentiation and mineralization of SCAPs.

### 2.4. Suppression of the BMP–Smad Signaling Pathway Reverses EGCG-Induced Osteo-/Odontogenic Differentiation of SCAPs

Several studies have reported that BMP–Smad signaling is critically involved in the regulation of the differentiation of MSCs [21,22]. Canonical BMP–Smad signaling leads to the induction of downstream transcription factors and odontogenic differentiation during root initiation and elongation [22,23]. Interestingly, in our study, the expression of BMP-2 as well as p-Smad1/5/9, which are two vital molecules in the BMP–Smad signaling pathway, were upregulated when EGCG was added, suggesting the potential role of the BMP–Smad signaling pathway in EGCG-mediated differentiation of SCAPs (Figure 4F).

**Figure 5:**
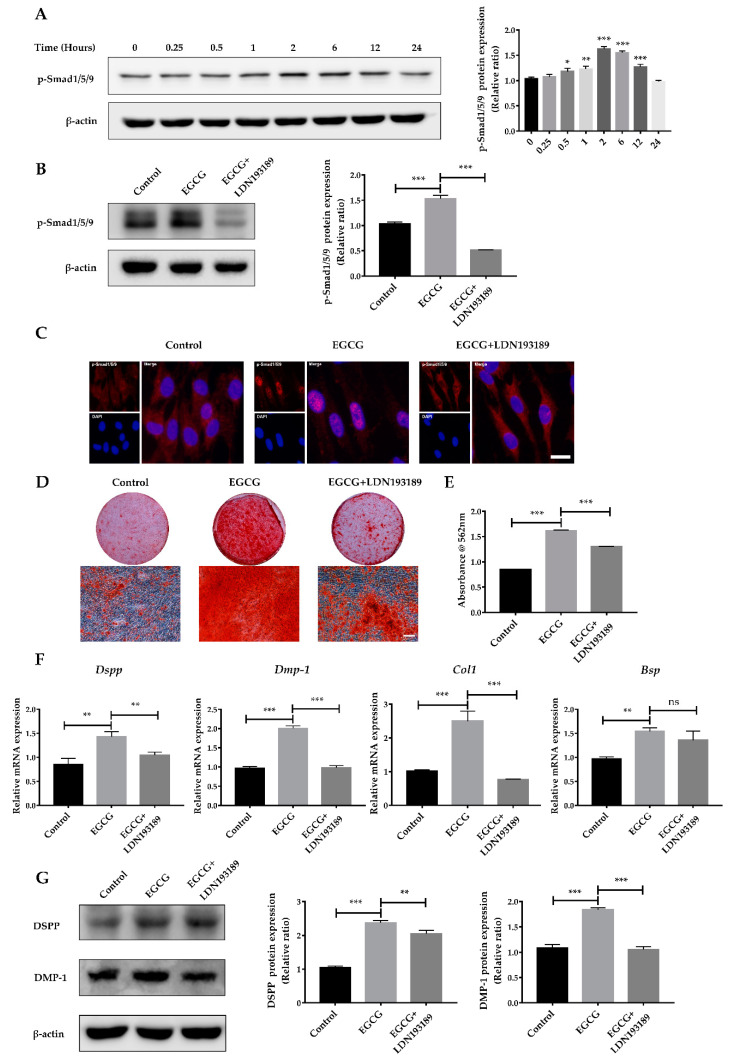
The BMP–Smad signaling pathway is involved in EGCG-stimulated osteo-/odontogenic differentiation. (**A**) The phosphorylation level of Smad1/5/9 in SCAPs treated with EGCG at different time points and the quantitative analysis. (**B**) Western blot analysis showing the increased phosphorylation of Smad1/5/9 was reversed by LDN193189. (**C**) Immunofluorescence staining indicating EGCG increased the translocation of p-Smad1/5/9 from the cytoplasm into the nucleus, but LDN193189 inhibited this transference. Scale bar = 20 μm. (**D**) ARS staining in the control (0 μM) group, EGCG (1μM) group, and EGCG+LDN193189 group after 14 days of induction. (**E**) Semi-quantification of calcium deposits. (**F**) The gene expression levels of *Dspp*, *Dmp-1*, *Bsp*, and *Col1* in inhibitor group (EGCG+LDN193189) were suppressed compared with in the EGCG group (*n* = 3). (**G**) The protein levels and quantitative analysis of DSPP and DMP-1 at 14 days. Error bars: mean ± standard deviation. (* *p* < 0.05, ** *p* < 0.01, *** *p* < 0.001, ns: not statistically significant).

Therefore, in order to determine whether the BMP–Smad signaling pathway was affected by EGCG treatment, SCAPs were treated with EGCG for 0.25, 0.5, 1, 2, 6, 12, or 24 h, respectively. p-Smad1/5/9, the important effector of BMP–Smad signaling, was significantly upregulated in SCAPs after EGCG treatment for at least 0.5 h (Figure 5A). These findings indicate that the BMP–Smad signaling pathway may be the downstream mechanism involved in the EGCG-induced osteo-/odontogenic differentiation of SCAPs.

To confirm whether BMP–Smad signaling is involved in the EGCG-induced osteo-/odontogenic differentiation of SCAPs, we employed LDN193189, an inhibitor of type I BMP receptor, to suppress BMP–Smad signaling. As shown in Figure 5B, LDN193189 (500 nM) treatment significantly suppressed the elevated phosphorylation level of Smad1/5/9 caused by EGCG stimulation. Notably, immunofluorescence staining identified the translocation of p-Smad1/5/9 from the cytoplasm into the nucleus with the stimulation of EGCG. However, the nuclear localization of p-Smad1/5/9 was decreased in the EGCG+LDN193189 group compared with the EGCG group (Figure 5C). Furthermore, LDN193189 significantly abrogated the effects of EGCG on the osteo-/odontogenic differentiation of SCAPs. After 14 days of osteo-/odontogenic differentiation, LDN 193,189 reversed EGCG-induced mineralized nodule formation, as indicated by ARS staining (Figure 5D,E). The expression levels of *Dspp*, *Dmp-1*, and *Col 1* were remarkably decreased in the EGCG+LDN193189 group compared with the EGCG group (Figure 5F). In addition, the protein levels of DSPP and DMP-1 showed a similar tendency to gene expression levels in SCAPs treated with LDN193189 (Figure 5G). Based on these results, we determined that BMP–Smad signaling is required for the EGCG-induced osteo-/odontogenic differentiation of SCAPs.

### 2.5. EGCG Shows the Comparable Ability to Promote Mineralization with Recombinant BMP2

**Figure 6:**
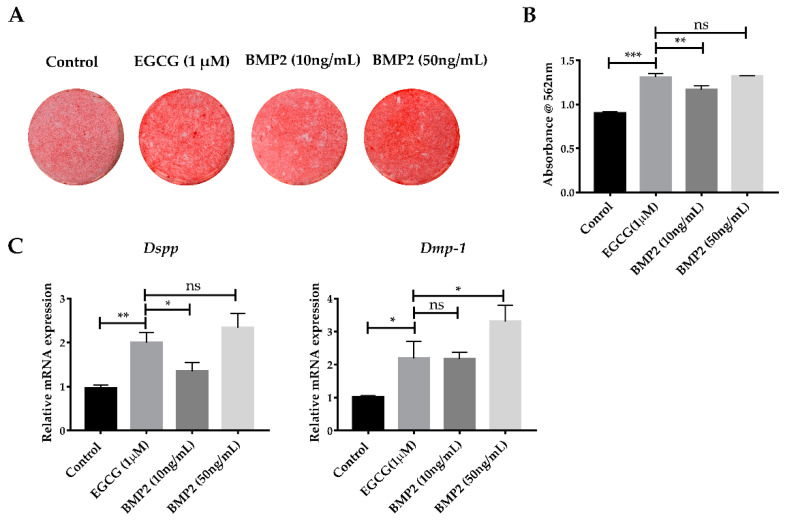
EGCG showed the ability to promote mineralization comparable with that of BMP2. (**A**) After 14 days of induction, the EGCG (1 μM) group generated amounts of calcified nodules similar to the 50 ng/mL BMP2 group. (**B**) Semi-quantification of mineralized deposits. (**C**) RNA expression of *Dspp* and *Dmp-1* at 14 days (*n* = 3). Error bars: mean ± standard deviation. (* *p* < 0.05, ** *p* < 0.01, *** *p* < 0.001, ns: not statistically significant).

BMP2 has shown a strong and reliable effect in promoting the osteo-/odontogenic differentiation of several kinds of stem cells, both in vitro and in vivo [20]. To facilitate a comprehensive understanding of the ability of EGCG to promote odontogenic differentiation, we compared the effective level of EGCG in osteo-/odontogenic differentiation and mineralization of SCAPs with that of BMP2 in vitro. Cells were treated with EGCG (1 μM) or recombinant BMP2 (10, 50 ng/mL). ARS staining indicated the mineralization of the EGCG-treated group had a comparable result with the 50 ng/mL BMP-treated group after 14 days of induction (Figure 6A,B). The RNA levels of *Dspp* showed expression levels comparable with the 50 ng/mL BMP-treated group, while *Dmp-1* exhibited a similar change between the EGCG-treated group and 10 ng/mL BMP-treated group (Figure 6C). These results suggested the EGCG might possess an ability to promote mineralization comparable with that of BMP2.

## 3. Discussion

In our study, we found that EGCG promotes the proliferation of SCAPs while showing no significant influence on cell migration at concentrations below 10 μM. Meanwhile, treatment with 1 or 10 μM EGCG enhances the osteo-/odontogenic differentiation of SCAPs as indicated by ALP activity, mineral deposition, and the expression of osteo-/odontogenic related markers. We further determined that EGCG promotes osteo-/odontogenic differentiation by activating the BMP–Smad signaling pathway in vitro. Finally, we found that EGCG shows an ability to promote mineralization comparable to that of recombinant BMP2. Our findings indicated that EGCG is an efficient natural chemical promoting the osteo-/odontogenic differentiation and mineralization of SCAPs and may be used for regenerative dentistry.

Dental tissue engineering requires stem/progenitor cells. Among the mesenchymal stem cells identified from dental tissues, SCAPs derived from the apex of an immature tooth may be a superior source for dentin regeneration due to their origin. In our study, we isolated and characterized human SCAPs, as the cells showed colony formation ability, multilineage differentiation potential, and the expression of mesenchymal stem cell markers (e.g., CD44, CD90, and CD146). Meanwhile, CD24, which might be a specific marker of SCAPs [6], can also be detected in the cells we isolated. However, the percentage of cells expressing CD24 shows a wide range [6,24]. This may be related with the donors or the cell condition, etc. SCAPs show significantly higher telomerase activity and express greater abundance of survivin than DPSCs [6]. This indicates that SCAPs might be more naive cells. Additionally, it has been documented that SCAPs express more DMP-1 than stem cells from human exfoliated deciduous teeth (SHED) under differentiation conditions, and genome-wide gene expression profiles show that SCAPs are precursors for primary odontoblasts, whereas SHED differentiate into replacement odontoblasts [25]. Meanwhile, SCAPs have a higher population doubling capacity along with higher proliferation and migration capacity when compared with DPSCs [6,26]. SCAPs also show higher mineralization potential and dentin regeneration capacity than DPSCs in vitro [26] and in vivo [6]. These important advantages make SCAPs a promising source for dental tissue regeneration.

In order to promote the efficiency of tissue regeneration, various molecules are used. In our study, we determined the dual effect of EGCG on the proliferation of SCAPs, that is, low concentrations of EGCG promote cell proliferation, while high concentrations of EGCG show an inhibited effect. Several studies have demonstrated that EGCG could promote the proliferation of cells such as PDLCs and human BMSCs in vitro at concentrations below 20 μM [11,12], which is consistent with our results. Differences in cell types, culture conditions, or detection methods may be accountable for the discrepancies in concentrations of EGCG. Nevertheless, previous studies have shown that higher concentrations of EGCG significantly suppress cell proliferation in cancer cells [27,28] and adult stem cells [16], similarly to our results. The inhibitive effect of EGCG at high concentrations may be related to apoptotic cell death via DNA damage and reduction of DNA synthesis [29]. On the other hand, cell migration has also been shown to be important in regenerative dentistry. High concentrations (25 and 50 μM) of EGCG may reduce the migration of human alveolar bone cells [30]. Therefore, a combination of molecules that promote migration like SDF-1α might be an option when we use higher concentrations of EGCG. Collectively, our findings showed that EGCG can promote the proliferation of SCAPs, and low concentrations (0.1 or 1 μM) of EGCG do not have a significant influence on cell migration. Therefore, 1–10 μM of EGCG may be the recommended concentration for future applications.

The regeneration of dentin involves the odontogenic differentiation of stem cells and the formation of mineralized tissues. Our results revealed that EGCG enhances the expression of osteo-/odontogenic markers including *BSP* and *Col 1* in SCAPs. Previous research reported that EGCG increases the expression of *Col1* and osterix (*Osx*) and promotes extracellular matrix mineralization in PDLCs [11]. Furthermore, several studies have found EGCG can enhance ALP activity, the expression of osteogenic markers like Runx2 and OCN, and the formation of mineralized nodules [10,11,31]. However, the effective concentrations for osteogenic differentiation varied among studies, which might be related to the cell types. Moreover, DMP-1 and DSPP, the two major phosphoproteins of non-collagenous proteins, can regulate dentin mineralization. In our study, these two representative odontogenic markers were also upregulated when EGCG was added. In accordance with previous studies, our findings indicate that EGCG can promote the osteo-/odontogenic differentiation and mineralization of SCAPs.

Although BMPs show a potent capacity to promote odontogenic differentiation and dentin regeneration, the large dose requirement and high cost are obstacles for clinical applications. It has been reported that an increase in BMP-2 has been identified in the EGCG-induced osteogenic differentiation of BMSCs [10]. In our study, we determined that EGCG increases the expression of BMP2 and the phosphorylation level of Smad1/5/9, and the suppression of BMP–Smad signaling using LDN193189 significantly attenuates the EGCG-induced osteo-/odontogenic differentiation of SCAPs. Moreover, we compared the effects of BMP2 and EGCG on the osteo-/odontogenic differentiation of SCAPs and determined the concentrations of BMP2 and EGCG, which, to some extent, exhibited similar capacities. Furthermore, recent studies reported that an EGCG-modified scaffold exhibited desirable morphological and mechanical characteristics, as well as having an anti-inflammatory effect, and could modulate the recruitment of cells [32,33,34]. EGCG is, therefore, a potential therapeutic molecule for tissue regeneration when incorporated with a scaffold or other agents. Further work using such an EGCG-modified scaffold with SCAPs in an animal model is still needed.

## 4. Materials and Methods

### 4.1. Cell Culture

For the harvesting of SCAPs, healthy immature human third molars were collected from patients aged 16–20 years (*n* = 5) according to the previous studies [6]. All protocols in the present work were approved by the Ethics Committee of the School of Stomatology, Wuhan University, China. In brief, apical papilla tissues were gently separated from teeth, thoroughly rinsed with phosphate buffered saline (PBS, Hyclone, Logan, UT), minced, and digested with 3 mg/mL type I collagenase (Biosharp, Hefei, China) and 4 mg/mL dispase (Roche, Mannheim, Germany) at 37 °C for 40 min. The digested mixtures were passed through a 70 μm cell strainer to get single-cell suspensions. SCAPs were cultured in the α-MEM (Hyclone) containing 10% fetal bovine serum (FBS, Gibco, Thornton, NSW, Australia) and 100 U/mL penicillin–streptomycin (Hyclone). Cells were incubated at 37 °C in an atmosphere with 5% CO_2_. SCAPs at passages 2 to 5 were used in this study.

### 4.2. Colony-Forming Assay

To characterize SCAPs, single-cell suspensions of SCAPs were seeded in a 10 cm dish. Cells were cultured in the culture medium for 10 days. After fixation in 4% paraformaldehyde (PFA), cells were immersed in crystal violet staining solution for 10 min (Beyotime, Shanghai, China). Clusters with more than 50 cells were considered as colonies.

### 4.3. Flow Cytometric Analysis

SCAP aliquots (1 × 10^6^ cells) were used to detect the immunophenotype according to a previously developed protocol [35]. The following anti-human monoclonal antibodies were used: CD45/FITC (Cat#304005, BioLegend, San Diego, CA, USA), CD34/FITC (Cat# 343603, BioLegend), CD90/FITC (Cat#328107, BioLegend), CD146/FITC (Cat#361011, BioLegend), CD44/FITC (Cat#338803, BioLegend), and CD24/PE (Cat#311105, BioLegend). Non-specific IgG/FITC (Cat#400107, BioLegend) or IgG/PE (Cat#400211, BioLegend) was used to be the isotype control. The data were analyzed using a FACS Calibur flow cytometer (Becton Dickinson, Franklin Lakes, NJ, USA).

### 4.4. Immunofluorescence Staining

For immunofluorescent analyses, cells were seeded on coverslips. After washing and fixation, cells at passage 2 were incubated with anti-Nestin (1:100, Santa Cruz Biotechnology, Santa Cruz, CA, USA), and cells treated with EGCG alone or EGCG combined with LDN193189 were incubated with anti-p-Smad1/5/9 (1:500, Cell Signaling Technology, Beverly, MA, USA) overnight. After one hour of incubation with Cy3-conjugated secondary antibodies (Jackson ImmunoResearch, West Grove, PE, USA), samples were counterstained with 4,6-diamidino-2-phenylindole (DAPI). Then, cells were photographed under a fluorescence microscope (Leica, Wetzlar, Germany).

### 4.5. Multiple Lineage Differentiation

For osteo-/odontogenic differentiation, SCAPs were cultured in six-well plates. The osteo-/odontogenic differentiation induction medium was supplied for three weeks. The induction medium contained α-MEM with 10% FBS, 50 mg/mL ascorbic acid, 10 mM sodium β-glycerophosphate, and 10 nM dexamethasone (Sigma-Aldrich, St. Louis, MI, USA) [36]. Cells were fixed with 95% ethanol, and then washed and stained with 1% Alizarin Red S (Sigma-Aldrich; pH = 4.2) in order to detect the formation of mineral deposits.

For adipogenic differentiation, cells were cultured in adipogenic medium (Cyagen Biosciences Inc, Sunnyvale, CA, USA) according to the manufacturer’s instructions. After four weeks, cells were fixed in 4% PFA. Next, Oil Red O reagent (Cyagen) was used to stain cells for 15 min to observe lipid droplets.

### 4.6. CCK-8 Assay

For the CCK-8 assay, 5 × 10^3^ SCAPs per well were seeded in 96-well plates and incubated with culture medium containing 0, 0.1, 1, 10, 20, 50, or 100 μM EGCG (Sigma-Aldrich, #E4143). The culture medium was renewed every other day. The proliferation of the SCAPs was measured with a CCK-8 assay kit (Dojindo, Kumamoto, Japan) after one, three, or five days of culture following the manufacturer’s instructions.

### 4.7. Wound Healing Assay

Wound healing assay was performed to assess the role of EGCG on the motility of SCAPs in vitro [8]. SCAPs were seeded in six-well plates, and the monolayers were scratched using a 200 μL pipette tip. PBS washed twice to remove cell debris. Cells were maintained in α-MEM containing 1% FBS and different concentrations of EGCG (0, 0.1, 1, 10, 20, or 50 μM). Images were captured immediately after scratching under a phase contrast microscope (Olympus, Tokyo, Japan). After 24 h, cells were fixed with 4% PFA. Then, cells were incubated with 0.1 mg/mL acridine orange and visualized under a fluorescent microscope (Olympus). Cells from five random areas were used to calculate the average amount of migrating cells in each group.

### 4.8. Alkaline Phosphatase (ALP) Staining and Alizarin Red S (ARS) Staining

Cells were cultured in 12-well plates supplied with osteo-/odontogenic differentiation induction medium. In different groups, EGCG was added into the medium at concentrations of 0, 0.1, 1, or 10 μM. The medium was renewed every three days. For ALP staining, after seven days of osteo-/odontogenic induction, cells from each group were fixed with 4% PFA. Then, cells were stained with CIP/NBT Alkaline Phosphatase Color Development Kit (Beyotime). The quantitation of ALP activity was detected using an Alkaline Phosphatase Assay Kit (Nanjing Jiancheng Bioengineering Institute, Nanjing, China) according to the manufacturer’s instructions. The quantitative values were measured at 520 nm absorbance. The values of ALP activity were calculated in U/gprot.

For ARS staining, cells were stained with 1% Alizarin Red S after 14 days of osteo-/odontogenic induction. For semi-quantification of ARS staining, 10% (*w*/*v*) cetylpyridinium chloride was added to the plates to destain the samples. The absorbance was determined at 562 nm using a microplate reader.

### 4.9. Quantitative Reverse Transcriptase Polymerase Chain Reaction (qRT-PCR) Analysis

The mRNA expression levels of *Dspp*, *Dmp-1*, *Bsp*, and *Col1* were determined via qRT-PCR. Briefly, total RNAs were isolated from SCAPs with the HP Total RNA Kit (Omega bio-tech, Norcross, GA, USA) after seven or 14 days of osteo-/odontogenic induction. Then, a Revert Aid First Strand cDNA Synthesis Kit (ThermoFisher Scientific, Rockford, IL, USA) was used to synthesize cDNA. The cDNA was then used for qRT-PCR in a CFX Connect Real-Time System (Bio-rad, Hercules, CA, USA) with SYBR Green Master (Roche). The expression levels of the target genes were normalized to GAPDH using the 2^−ΔΔCt^ method. The primers for qRT-PCR are listed in Table 1.

### 4.10. Western Blotting

SCAPs treated with EGCG, LDN193189 (Sigma-Aldrich), or recombinant human BMP2 (PeproTech, Rocky Hill, NJ) at different time points were lysed in lysis buffer (Beyotime), and the protease inhibitor cocktail (MCE, Shanghai, China) was added. After centrifuging at 12,000 rpm for 10 min at 4 °C, total protein was collected from the lytic cells. The concentrations of proteins were measured using a BCA Kit (Thermo). Equal amounts of proteins were loaded onto 10% SDS-polyacrylamide gel. The samples were separated by electrophoresis and then transferred onto PVDF membranes (Roche Diagnostics GmbH, Germany). The following primary antibodies were used: DSPP (Novus, Centennial, Colorado; Cat#NBP1-91612, 1:1000), DMP-1 (Abcam, Cambridge, UK; Cat#103203, 1:1000), p-Smad1/5/9 (Cell Signaling Technology; Cat#13820, 1:1000), β-actin (MBL, Tokyo, Japan; Cat#PM053-7, 1:6000), and BMP2 (Abcam; Cat#6285, 1:1000). The target bands were quantified using Image J and normalized to β-actin.

### 4.11. Statistical Analysis

The results shown in mean ± standard deviation were representative of three independent experiments. The equal variance between individual groups showed no significant difference. The data in the study were analyzed using one-way analysis of variance (ANOVA), followed by Tukey’s multiple post hoc tests using GraphPad Prism 7.0 (GraphPad Software, La Jolla, CA, USA). *p* < 0.05 was considered statistically significant.

## 5. Conclusions

Based on the findings of our study, we found that EGCG promotes the proliferation of SCAPs, and low doses (0.1 or 1 μM) of EGCG do not influence the migration of SCAPs. EGCG increases the osteo-/odontogenic differentiation of SCAPs by activating the BMP–Smad signaling pathway. EGCG may possess a comparable ability to promote mineralization with recombinant BMP2 in vitro. Our results suggest EGCG can be used as an effective natural compound in dentin regeneration.

## Figures and Tables

**Table 1:** Primers for qRT-PCR.

Genes	Forward Primer (5′-3′)	Reverse Primer (5′-3′)
*Gapdh*	TCATGGGTGTGAACCATGAGAA	GGCATGGACTGTGGTCATGAG
*Dspp*	TGCTGGAGCCACAAAC	AAACCCTATGCAACCTTC
*Dmp-1*	ACAGGCAAATGAAGACCC	TTCACTGGCTTGTATGG
*Bsp*	CGAAGCAGAAGTGGATGAAA	TGCCTCTGTGCTGTTGGTACTG
*Col1*	GCGGCTCCCCATTTTTATACC	GCTCTCCTCCCATGTTAAATAGCAC

Gapdh: glyceraldehyde-3-phosphate dehydrogenase; Dspp: dentin sialophosphoprotein; Dmp-1: dentin matrix protein-1; Bsp: bone sialoprotein; Col1: Type I collagen.

## Data Availability

The data presented in this study are available included in the article.

## Sample Availability

Not available.
